# Klinische Erfahrungen mit Cefiderocol: Neue Therapieoption bei schweren Infektionen durch multiresistente gramnegative Erreger

**DOI:** 10.1007/s00063-022-00925-5

**Published:** 2022-08-01

**Authors:** Oliver Witzke, Thorsten Brenner

**Affiliations:** 1 Klinik für Infektiologie, Westdeutsches Zentrum für Infektiologie, Universitätsmedizin Essen, Universität Duisburg-Essen, Hufelandstr. 55, 45122 Essen, Deutschland; 2 Klinik für Anästhesiologie und Intensivmedizin, Universitätsmedizin Essen, Universität Duisburg-Essen, Essen, Deutschland

**Keywords:** Kritisch kranke Patienten, Pseudomonas aeruginosa, 4MRGN, Enterobacterales, Siderophor-Cephalosporin, Critically ill patients, Pseudomonas aeruginosa, XDR, Enterobacterales, Siderophore-Cephalosporin

## Abstract

**Hintergrund:**

Infektionen mit antibiotikaresistenten Bakterien stellen eine hohe Gesundheitsbelastung dar, da sie mit erhöhter Letalität assoziiert sind und längerfristige dramatische Beeinträchtigungen der Lebensqualität hervorrufen können. In Deutschland erkranken jährlich etwa 54.500 Menschen an Infektionen durch antibiotikaresistente Erreger, von denen etwa 2400 Menschen versterben. Infektionen mit multiresistenten gramnegativen Bakterien (MRGN), insbesondere mit carbapenemresistenten Erregern, stellen ein besonderes Risiko dar, da nur eine begrenzte Zahl an Therapieoptionen verfügbar ist.

**Fragestellung:**

Wie sind die Ergebnisse aus Studien und Compassionate-Use-Programm mit dem neuen Siderophorantibiotikum Cefiderocol, das im April 2020 von der Europäischen Arzneimittel-Agentur (EMA) bei Erwachsenen zur Behandlung von Infektionen durch aerobe gramnegative Erreger zugelassen wurde, wenn nur begrenzte Behandlungsmöglichkeiten zur Verfügung stehen? Die Zulassung ist pathogenbasiert und fokusunabhängig [5].

**Ergebnisse:**

Cefiderocol, das über einen innovativen Zelleintrittsmechanismus verfügt, ist als erstes β‑Laktam-Antibiotikum aus der Gruppe der Cephalosporine stabil gegenüber allen klinisch relevanten β‑Laktamasen, einschließlich Carbapenemasen, und hat eine hohe In-vitro-Wirksamkeit gegenüber carbapenemresistenten MRGN. Die Ergebnisse werden durch klinische Studien bei komplizierten Harnwegsinfektionen, nosokomialer Pneumonie/Beatmungspneumonie und schweren Infektionen durch carbapenemresistente Erreger bestätigt.

**Fazit:**

Klinische Studiendaten sowie die Ergebnisse aus den weltweiten Erfahrungsberichten zeigen, dass Cefiderocol eine vielversprechende Behandlungsoption für schwere Infektionen durch multiresistente, insbesondere carbapenemresistente gramnegative Bakterien darstellt.

## Abstract (en)

**Background:**

Infections due to antibiotic-resistant bacteria are threatening modern healthcare, and antibacterial resistance has become one of the greatest threats to public health. In Germany 54,500 patients become infected with antibiotic-resistant bacteria per year, causing about 2400 attributable deaths. Rising resistance in Gram-negative bacteria especially carbapenem-resistant pathogens is of particular concern due to the lack of effective and safe alternative treatment options.

**Objective:**

The results from trials and compassionate-use programs with the new antibiotic cefiderocol, which was approved by the European Medicines Agency (EMA) in April 2020 for the treatment of adults with infections caused by aerobic Gram-negative bacteria, are summarized.

**Results:**

The new β‑lactam antibiotic cefiderocol is the first siderophore cephalosporin indicated for the treatment of infections due to aerobic Gram-negative organisms in adults with limited treatment options. Its chemical structure and its unique mechanism of action confer enhanced stability against β‑lactamases including all classes of clinically relevant carbapenemases. In vitro data show high antibacterial activity against multidrug resistant Gram-negative bacteria, Enterobacterales and nonfermenters, including carbapenem-resistant strains. In clinical trials, cefiderocol showed superiority in complicated urinary tract infection in comparison to imipenem and non-inferiority versus meropenem in hospital-acquired/ventilator-associated pneumonia patients and severe infections caused by carbapenem-resistant pathogens.

**Conclusion:**

Clinical trial data and case reports identified in the literature search show that cefiderocol is a promising treatment option for severe infections caused by drug-resistant Gram-negative bacteria, particularly carbapenem-resistant bacteria.

## Hintergrund

Multiresistente gramnegative Bakterien (MRGN), deren Inzidenz seit Jahren zunimmt, können schwere Infektionen mit hoher Morbidität und Letalität verursachen, da routinemäßig eingesetzte und klinisch etablierte Antibiotika häufig nicht mehr ausreichend wirksam sind [2, 4]. Besonders kritisch ist der Resistenzanstieg gegenüber Carbapenemen, die lange Zeit eine sichere (oftmals letzte) Option bei schweren MRGN-Infektionen waren. Die Weltgesundheitsorganisation (WHO) hat Infektionen durch carbapenemresistente *Enterobacteriaceae* (Enterobacterales/CRE), *Pseudomonas aeruginosa* und *Acinetobacter baumannii* daher als besonders kritisch bewertet [23].

## Carbapenemresistente Enterobacterales (CRE), *Pseudomonas aeruginosa* und *Acinetobacter baumannii*

Wie Veröffentlichungen des Robert Koch-Instituts (RKI) zeigen, hat sich auch in Deutschland die Zahl der Infektionen durch carbapenemresistente gramnegative Bakterien im Verlauf der Jahre deutlich erhöht [9, 18]. Multiresistente gramnegative Bakterien verfügen über eine Vielzahl an unterschiedlichen Resistenzmechanismen. Hierzu gehören diverse β‑Laktamasen, Veränderungen der Porinkanäle sowie bakterielle Effluxpumpen, die das Antibiotikum aktiv aus der Bakterienzelle herauspumpen können [3, 4]. Wenngleich die antibiotische Pipeline eine Reihe an neuen Substanzen hervorgebracht hat, die bestimmte multiresistente GN-Erreger besser erfassen (z. B. Ceftolozan-Tazobactam für multiresistente *Pseudomonas aeruginosa* und Ceftazidim-Avibactam für KPC- und „OXA-48-like“ produzierende Enterobacterales), bestehen weiterhin Lücken bei Erregern, die Metallocarbapenemasen (NDM, VIM, IMP u. a.) produzieren, oder solchen, die multiple Resistenzmechanismen vorweisen (Tab. 1). Gleiches gilt für die neuen β‑Laktam-β-Laktamase-Inhibitor-Kombinationen Meropenem-Vaborbactam und Imipenem-Relebactam (Tab. 1; [8]). Zunehmend Probleme bereiten Carbapenem-Resistenzen, die nicht Carbapenemase-bedingt sind wie Mutationen, die wichtige Porinkanäle in der äußeren Membran verschließen oder eine Überproduktion von Effluxpumpen. Das Wirkspektrum der Cephalosporin-Inhibitor-Kombinationen umfasst zudem nicht *Stenotrophomonas maltophilia* und *Burkholderia cepacia*, die ebenfalls mit hohen Resistenzen gegenüber vielen Antibiotika assoziiert sind. Mit Cefiderocol steht ein neues β‑Laktam-Antibiotikum aus der Gruppe der Cephalosporine zur Verfügung, das diese Resistenzmechanismen überwindet und die bestehenden Wirklücken schließen kann (Tab. 1; [8, 26]).

**Table 1:** 

	In-vitro-Wirksamkeit							
	Enterobacteriaceae/Enterobacterales			*P. aeruginosa*	*A. baumannii*	*S. maltophilia*	Indikationen (inkl. erwartete)	Erregerfokussierte Studie (inkl. erwartet)
Antibiotikum	Klasse-A-Carbapenemase (z. B. KPC)	Klasse-B-Carbapenemase (z. B. NDM)	Klasse-D-Carbapenemase (z. B. OXA-48)					
Ceftazidim-Avibactam	Ja	Nein	Ja	Ja	Nein	Nein	cUTI/AP, cIAI, HABP/VABP	Nein
Ceftolozan-Tazobactam	Nein	Nein	Nein	Ja	Nein	Nein	cUTI/AP, cIAI, NP	Nein
Meropenem-Vaborbactam	Ja	Nein	Nein	Nein^a^	Nein	Nein	cUTI/AP	Ja
Imipenem-Cilastatin-Relebactam	Ja	Nein	Nein	Ja	Nein	Nein	cUTI/AP, cIAI, HABP/VABP	Ja
Cefiderocol	Ja	Ja	Ja	Ja	Ja	Ja	cUTI/AP, HABP/VABP	Ja
Fosfomycin	Ja	Ja	Ja	Variabel	Nein	Nein	cUTI/AP	Nein

*A. baumannii* *Acinetobacter baumannii*,* AP* akute Pyelonephritis, *cIAI* komplizierte intraabdominelle Infektion, *cUTI* komplizierte Harnwegsinfektion, *HABP* nosokomiale bakterielle Pneumonie, *KPC* Klebsiella-pneumoniae-Carbapenemase, *NDM* New-Delhi-Metallo-β-Laktamase, *NP* nosokomiale Pneumonie, *OXA* Oxacillinase, *P. aeruginosa* *Pseudomonas aeruginosa*, *S. maltophilia* *Stenotrophomonas maltophilia*, *VABP* beatmungsassoziierte bakterielle Pneumonie

^a^Nicht wirksam im Vergleich zu Meropenem allein

## Wirkmechanismus von Cefiderocol und Überwindung bakterieller Resistenzmechanismen

Das neue Cephalosporin Cefiderocol ist der erste zugelassene Vertreter der neuartigen Siderophorantibiotika mit einer Katecholeinheit und verfügt aufgrund seiner chemischen Struktur über einen einzigartigen Zelleintrittsmechanismus. Bakterien haben einen natürlichen Eisenbedarf für intrazelluläre Stoffwechselprozesse und können endogene Siderophore aussenden, um extrazelluläres 3‑wertiges Eisen aufzunehmen. Die Katecholgruppe des Cefiderocolmoleküls erfüllt diese Funktion als ein eisenbindendes Siderophor, sodass Cefiderocol mithilfe eines aktiven bakteriellen Fe^3+^-Transportsystems aktiv in das Zellinnere gramnegativer Bakterien eingeschleust werden kann und hohe Cefiderocolkonzentrationen im periplasmatischen Raum erzielt werden. Ungehindert kann es an die in der Zytoplasmamembran lokalisierten penizillinbindenden Proteine (PBP) binden, dadurch die Zellwandsynthese inhibieren und den Bakterienzelltod herbeiführen. Cefiderocol verhält sich somit wie ein „Trojanisches Pferd“. Aufgrund des siderophorvermittelten Einschleusens von Cefiderocol in die Zelle haben ein Porinverlust oder eine effluxvermittelte Resistenz bei Cefiderocol generell geringere Auswirkungen auf die In-vitro-Aktivität als bei vielen anderen β‑Laktam-Antibiotika. Durch Seitenketten in Position 3 und in Position 7 wird darüber hinaus eine besonders hohe Stabilität gegenüber β‑Laktamasen einschließlich Serin (KPC, OXA), Metallocarbapenemasen (NDM, VIM, IMP) und Carbapenemasen erzielt [8, 11, 25]. Internationale Surveillance-Studien belegen eine hohe In-vitro-Wirksamkeit von Cefiderocol gegenüber einem breiten Spektrum gramnegativer, aerober Infektionserreger einschließlich carbapenemresistenter Stämme. Hierzu gehören Enterobacterales (z. B. *Klebsiella* spp., *E. coli, Enterobacter* spp., *Proteus* spp. *Morganella *spp., *Citrobacter* spp., *Serratia* spp.), weiterhin die Nonfermenter *Pseudomonas aeruginosa, Acinetobacter baumannii, Burkholderia cepacia* und *Stenotrophomonas maltophilia* [5, 7, 10, 20].

Aufgrund zunehmender Resistenzen bei gramnegativen Erregern erfüllt Cefiderocol einen wichtigen „medical need“ bei

- MRGN mit Effluxpumpenhochregulation und Porinkanalveränderungen;
- Metallo-β-Laktamase‑, KPC- und vielen OXA-Carbapenemase-Produzenten;
- multiresistenten Erregern einschließlich 4MGRN, wie *Pseudomonas aeruginosa, Acinetobacter baumannii* und *Klebsiella pneumoniae*;
- intrinsisch β‑Laktam-resistenten *Stenotrophomonas maltophilia*.

## Dosierung und PK/PD bei kritisch kranken Patienten

Kürzlich publizierte Daten weisen darauf hin, dass die Dosierungsempfehlungen für Cefiderocol [5] zu angemessenen Spiegeln im Plasma und in der Epithelflüssigkeit führen. König et al. untersuchten jetzt die PK/PD-Eigenschaften der Substanz bei kritisch kranken Patienten mit septischem Schock und Nierenversagen (einschließlich kontinuierlicher Nierenersatztherapie und Zytokinadsorbertherapie), die mit Cefiderocol unter Bedingungen des klinischen Alltags behandelt wurden. Ursächliche Erreger waren multiresistente *Pseudomonas aeruginosa* und multiresistente *Acinetobacter baumannii*. Die Dosierungsanweisungen des Herstellers gemäß Fachinformation waren ausreichend, um einen Wirkstoffspiegel deutlich über der MHK dieser Erreger zu erreichen. Der Zusatz eines Zytokinadsorbers könnte jedoch die Serumspiegel erheblich senken, sodass in diesem Zusammenhang eine therapeutische Überwachung und eine Dosisanpassung empfohlen werden. Im Rahmen dieser Untersuchung konnte trotz der besonderen Schwere der Erkrankung bei 60 % der Patienten ein mikrobiologischer bzw. klinischer Erfolg erzielt werden [12].

## Klinische Wirksamkeit in Phase-III-Zulassungsstudien

### Komplizierte Harnwegsinfektionen (APEKS-cUTI) durch gramnegative Erreger mit oder ohne Pyelonephritis

Im Rahmen der APEKS-cUTI (*Acinetobacter baumannii, Pseudomonas aeruginosa, Escherichia coli, Klebsiella pneumoniae* und *Stenotrophomonas maltophilia* bei Patienten mit komplizierten Harnwegsinfektionen) einer multizentrischen randomisierten Doppelblindstudie wurde die klinische Wirksamkeit von Cefiderocol (3-mal 2 g als Infusion über eine Stunde) bei Patienten mit komplizierten Harnwegsinfektionen (cUTI) durch gramnegative Erreger und/oder akuter unkomplizierter Pyelonephritis (AUP) bei 371 Patienten (MITT-Population) untersucht.

Die Studie war statistisch darauf ausgelegt, die Nichtunterlegenheit von Cefiderocol nachzuweisen. Eine Post-hoc-Analyse zeigte die Überlegenheit von Cefiderocol, das der Vergleichsmedikation Imipenem/Cilastatin sowohl hinsichtlich des primären Zielkriteriums (klinischer und mikrobiologischer Erfolg bei der MITT-Population) als auch bezüglich des sekundären Zielkriteriums (mikrobiologischer Erfolg beim Zeitpunkt „test of cure“ in der MITT-Population) überlegen war [19].

### Nosokomiale Pneumonie und Beatmungspneumonie APEKS-NP

**Figure 1:**
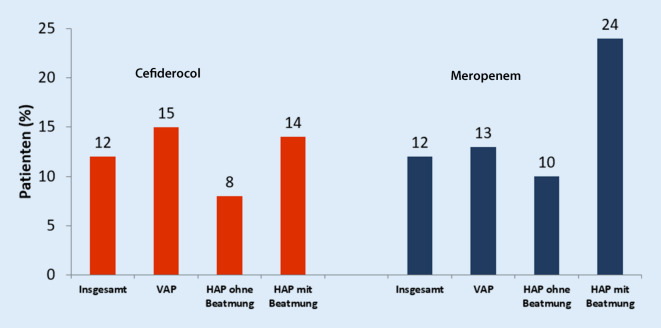

Die Wirksamkeit von Cefiderocol bei nosokomialer Pneumonie (HAP) und ventilatorassoziierter Pneumonie (VAP) durch gramnegative Erreger wurde in der APEKS-NP-Zulassungsstudie, einer internationalen multizentrischen randomisierten Nichtunterlegenheitsdoppelblindstudie, klinisch geprüft. Cefiderocol (2 g als prolongierte Infusion über 3 h alle 8 h) wurde mit einer Meropenemhochdosistherapie (3-mal 2 g) als „extended infusion“ ebenfalls über 3 h verglichen, um nach Expertenbewertung die Meropenemwirkung bei schwer kranken Patienten zu optimieren. Im Gegensatz dazu waren die HAP/VAP-Zulassungsstudien REPROVE (mit Ceftazidim-Avibactam) und ASPECT-NP (mit Ceftolozan-Tazobactam) gegen jeweils nur 1 g Meropenem (3-mal täglich) als Kurzinfusion über 30 bzw. 60 min durchgeführt worden [13, 21, 24]. Die Cefiderocolbehandlung und die Meropenemhochdosisbehandlung erfolgten für 7–14 Tage. Linezolid wurde in beiden Armen für mindestens 5 Tage verabreicht, um methicillinresistente *Staphylococcus aureus* (MRSA) und andere grampositive Bakterien abzudecken. Insgesamt wurden 300 Patienten in die Studie aufgenommen (Cefiderocol *n* = 148, Meropenem *n* = 152), von denen ungefähr zwei Drittel aus Europa stammten. 60 % der Patienten wurden maschinell beatmet; 68 % waren Intensivpatienten bei Randomisierung und 49 % hatten einen APACHE-II-Score ≥ 16 [24]. Häufigste Erreger waren *Klebsiella pneumoniae, Pseudomonas aeruginosa, Acinetobacter baumannii, E. coli* und *Enterobacter cloacae*. Der Anteil der mITT-Patienten mit dem Infektionsstatus „schwer“ war höher in der Cefiderocolgruppe (49 %) im Vergleich zur Meropenemgruppe (33 %). Cefiderocol erreichte den primären Endpunkt der Nichtunterlegenheit im Vergleich zu einer Meropenemhochdosistherapie (verabreicht als „extended infusion“) bezüglich der *14-Tage-Gesamtmortalität* in der mITT-Population nach Beginn der Studienmedikamentengabe. Bei HAP-Patienten mit Beatmung lag die Gesamtmortalität in der Cefiderocolgruppe mit 14 % numerisch niedriger als in der Hochdosismeropenemgruppe mit „extended infusion“ (24 %; Abb. 1; [24]).

**Figure 2:**
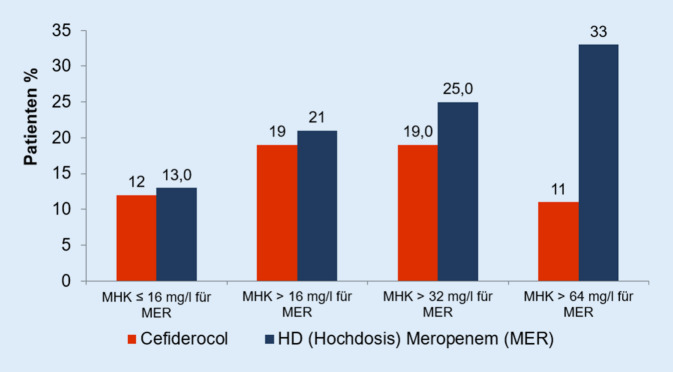

Die Gesamtmortalität an Tag 28 (21,0 % vs. 21,0 %) war vergleichbar in beiden Gruppen, ebenso die klinischen Heilungsraten (65 % vs. 67 %; [24]). Unter Berücksichtigung der mikrobiologisch auswertbaren Patienten (ME-Population) lag die Gesamtmortalität bei 12,4 % in der Cefiderocolgruppe und bei 13 % in der Meropenemgruppe [16]. Eine weitere Auswertung berücksichtigte nur Patientendaten, bei denen ein Erreger isoliert wurde und zusätzlich die minimale Hemmkonzentration (MHK) bestimmt wurde (Abb. 2).

Bei Infektionen durch Erreger mit Meropenemresistenz und höheren Meropenem-MHK-Werten blieben sowohl die Erregereradikationsraten als auch die Gesamtmortalität unter der Cefiderocolbehandlung gleich, wohingegen unter der Meropenembehandlung die Eradikationsraten zurückgingen und die Gesamtletalität anstieg (Abb. 2; [24]).

APEKS-NP ist die bisher einzige Vergleichsstudie gegen Hochdosismeropenem mit verlängerter Infusionszeit und belegt die klinische Wirksamkeit von Cefiderocol auch bei carbapenemresistenten gramnegativen Erregern [24].

## CREDIBLE-CR: schwere Infektionen mit carbapenemresistenten Erregern

CREDIBLE-CR ist eine deskriptive, nichtinferentielle, offene, nichtvergleichende, multizentrische, multinationale Studie bei schwerstkranken Patienten mit nosokomialer Pneumonie einschließlich ventilatorassoziierter Pneumonie (HAP/VAP/HCAP), komplizierten Harnwegsinfektionen (cUTI) oder Blutstrominfektionen (BSI) und Sepsis durch carbapenemresistente (CR) gramnegative Erreger. Die Patienten wurden in dieser erregerfokussierten Studie entweder mit Cefiderocol oder der besten verfügbaren Therapie („best available therapy“, BAT) behandelt. Da es sich um kritisch kranke Patienten handelte (vergleichbar mit Compassionate Use), konnten die behandelnden Ärzte bei BAT bis zu 3 verschiedene geeignete Antibiotika für gramnegative Erreger frei kombinieren. Bei Cefiderocol war nur ein weiteres, gegenüber gramnegativen Erregern wirksames Antibiotikum erlaubt [1].

Die überwiegende Mehrheit der Patienten in der Cefiderocolgruppe erhielt eine Cefiderocolmonotherapie (83 %), die überwiegende Mehrheit in der BAT-Gruppe (71 %) eine Kombinationstherapie, überwiegend basierend auf Colistin mit anderen Reserveantibiotika in Zweier- oder Dreierkombination (Tab. 2).

Primäres Zielkriterium war die klinische Heilungsrate beim Zeitpunkt „test of cure“ (TOC) bei Patienten mit HAP/VAP/HCAP, BSI/Sepsis und das mikrobiologische Ansprechen bei Patienten mit cUTI [1].

In den beiden Behandlungsgruppen gab es zum Zeitpunkt der Randomisierung beträchtliche Unterschiede (Tab. 2, oben). Im Vergleich zu den mit BAT behandelten Patienten war ein größerer Anteil der mit Cefiderocol behandelten Patienten ≥ 65 Jahre alt und befand sich bei Randomisierung auf der Intensivstation und/oder hatte einen septischen Schock (oder mit Vorgeschichte, 31 Tage). Weiterhin war die Verteilung der zugrunde liegenden Pathogene (*Stenotrophomonas maltophilia*) nicht ausgeglichen, da diese kein Kriterium der Stratifizierung darstellten.

34 (34 %) von 101 Patienten, die Cefiderocol erhielten, und 9 (18 %) von 49 Patienten in der BAT-Gruppe starben bis zum Ende der Studie; einer dieser Todesfälle (in der BAT-Gruppe) wurde mit dem Studienmedikament in Verbindung gebracht [1]. Die beobachtete Mortalität in der Cefiderocolgruppe war so, wie man sie in der Patientenpopulation erwarten würde und wie sie in anderen vergleichbaren Studien beobachtet wurde. Die in der BAT-Gruppe beobachtete Mortalität war jedoch auffällig niedriger als erwartet und/oder in anderen Studien beobachtet [1, 14]).

### Nonfermenter *Acinetobacter baumannii*

Subgruppenanalysen zeigten, dass der beobachtete Mortalitätsunterschied primär bei Infektionen mit Beteiligung von CR *Acinetobacter *spp. bestand.

In dieser an *Acinetobacter*-Infektionen erkrankten Patientengruppe gab es bei Randomisierung in der Cefiderocolgruppe mit 26 % einen höheren Anteil an Patienten mit septischem Schock (oder mit Vorgeschichte, 31 Tage) im Vergleich zu Patienten in der BAT-Gruppe mit 6 %. Des Weiteren befanden sich in der Cefiderocolgruppe 81 % dieser Patienten bei Randomisierung auf der Intensivstation im Vergleich zu nur 47 % in der BAT-Gruppe (Tab. 2; [1]).

### Nonfermenter *Pseudomonas aeruginosa*

Die Mortalität bei Infektionen mit CR *Pseudomonas aeruginosa* (ohne *Acinetobacter*-Beteiligung) war 13 % in der Cefiderocolgruppe vs. 22 % in der BAT-Gruppe (Tab. 2; [15]).

### CR Enterobacterales (CRE)

Die Gesamtmortalität bei CRE-Infektionen war niedriger bei Patienten, die mit Cefiderocol behandelt wurden (13,8 % [C] vs. 27,3 % [BAT]; Tab. 2; [14]).

**Table 2:** 

	Cefiderocolgruppe^1^	BAT-Gruppe^1^
**Patient auf ICU bei Einschluss**		
Alle	56 %^a^	43 %^a^
*Acinetobacter*-Infektionen	81 %^b^	47 %^b^
**Septischer Schock in Vorgeschichte oder bei Randomisierung**	19 %^a^	12 %^a^
	26 %^b^	6 %^b^
**Anzahl *Acinetobacter***	37	17
**Anzahl *Stenotrophomonas***	5	0
**Alter**	≥ 65: 63 %	≥ 65: 45 %
Antibiotikamonotherapie	83 %	29 %
Kreatininclearance < 30	20 %	14 %
Mortalität CRE^2^	13,8 %	27,3 %
Klinische Heilung CRE^1^	66 %	45 %
Klinische Heilung MBL-Bildner^3^	75 %	29 %
Mortalität bei *Pseudomonas*^4^	13 %	22 %
Klinische Heilung *Pseudomonas*^4^	75 %	55 %
Therapiebedingte Nebenwirk.^1^		
Alle	15 %	22 %
Schwere	1 %	10 %

*BAT* „best available therapy“, *CRE* carbapanemresistente Enterobacterales, *MBL* Metallo-β-Laktamasen

^a^Alle

^b^nur *Acinetobacter*-Infektionen

^1^ [1]

^2^ [14]

^3^ [1], Appendix

^4^ [15]

### Weitere Studienergebnisse

Alle Unterschiede waren numerisch und nicht signifikant.

Die klinische Heilung (primäres Zielkriterium der Studie) betrug in der Cefiderocolgruppe (C) 53 % und in der BAT-Gruppe (BAT) 50 %. Indikationsspezifische Heilungsraten bei HAP, VAP oder HCAP lagen bei 50 % (C) vs. 53 % (BAT), bei Blutstrominfektionen (BSI) oder Sepsis bei 43 % (C) vs. 43 % (BAT) und bei cUTI 71 % (C) vs. 60 % (BAT; [1]).

Bei Infektionen mit carbapenemresistenten Erregern (CRE) wurde eine höhere Heilungsrate in der Cefiderocolgruppe erzielt als in der BAT-Gruppe (66 % vs. 45 %). Bei Nonfermentern waren sie in beiden Gruppen ähnlich. Bei Infektionen mit Metallo-β-Laktamasen(MBL)-bildenden Erregern lagen die klinischen Heilungsraten bei 75 % in der Cefiderocolgruppe und bei 29 % in der BAT-Gruppe (Tab. 2; [1]).

### Fazit der Autoren

Die offene erregerfokussierte CREDIBLE-CR-Studie belegt die Wirksamkeit und Sicherheit von Cefiderocol bei schweren Infektionen bei Hochrisikopatienten durch carbapenemresistente gramnegative Erreger in einer sehr heterogenen Patientenpopulation mit meist komplexen Grunderkrankungen [1]. In den beiden Behandlungsgruppen gab es zum Zeitpunkt der Randomisierung beträchtliche Unterschiede (Tab. 2).

## Cefiderocolverträglichkeit

Die bisher ausgewerteten klinischen Studien und Daten aus dem klinischen Alltag bestätigen für Cefiderocol die vielfach dokumentierte gute Verträglichkeit der β‑Laktam-Antibiotika [5]. Dies ist gerade bei Patienten mit schweren Infektionen von besonderer Bedeutung, da diese oftmals multimorbide sind, besondere Risikofaktoren haben, mehrere Medikamente erhalten und häufig auf der Intensivstation behandelt werden. Nach Expertenbewertung sollte unter diesem Aspekt ein β‑Laktam-Antibiotikum bei gleicher In-vitro-Wirksamkeit vor allen anderen Antibiotikaklassen bevorzugt eingesetzt werden [22]. Cefiderocol bleibt trotz Siderophoranteil ohne Einfluss auf den Eisenstoffwechsel. Die Eisenhomöostase des Körpers wird nicht verändert [19]. Bei Patienten mit Nierenfunktionsstörung erfolgt eine Dosisanpassung [5].

## Compassionate-Use-Programm und weitere Ergebnisse aus dem klinischen Alltag

Der erfolgreiche Einsatz von Cefiderocol im klinischen Alltag wurde u. a. im Rahmen des Compassionate-Use-Programms dokumentiert [6, 26]. Behandelt wurden Patienten mit sehr schweren Infektionen durch multiresistente oder XDR Erreger:

- Aortenklappenendokarditis mit XDR *Pseudomonas aeruginosa* bei rektaler Kolonisation mit OXA-48 *Klebsiella pneumoniae* und OXA-23/OXA-51 *Acinetobacter baumannii*;
- Blutstrominfektionen und Beatmungspneumonien durch XDR *Acinetobacter baumannii* und carbapenemasebildende *Klebsiella pneumoniae*;
- schwere intraabdominelle Infektion durch multiresistente *Pseudomonas aeruginosa*;
- neutropenischer Patient mit chronischer Osteomyelitis durch multiresistente *Pseudomonas aeruginosa*;
- Polytraumapatient mit früher postoperativer implantatassoziierter Infektion mit *Acinetobacter baumannii* (NDM, OXA-40) ;
- Traumapatient mit nosokomialer Pneumonie durch *Klebsiella pneumoniae* und XDR *Acinetobacter baumannii*;
- Patienten mit polymikrobieller Osteomyelitis (nach mehreren operativen Débridements infolge eines Traumas) und schwerer XDR *Acinetobacter-baumannii*-Infektion.

Alle Patienten waren vorbehandelt, in erster Linie mit Kombinationen aus Colistin und einem Carbapenem und/oder anderen Antibiotika. Die Umstellung auf Cefiderocol erfolgte aufgrund mangelnden klinischen Ansprechens oder einer Verschlechterung unter der Kombinationstherapie bzw. aufgrund von Nebenwirkungen (z. B. Nierenversagen unter Colistin und Erhöhung der Leberparameter unter Aztreonam; [26]).

Pascale et al. berichten über eine multizentrische Beobachtungsstudie bei 107 Intensivpatienten mit schweren COVID-19-Infektionen und der Diagnose carbapenemresistente *Acinetobacter-baumannii*-Infektion, überwiegend Infektionen des Blutstroms und der unteren Atemwege. Alle 42 Patienten in der Cefiderocolgruppe (100 %) erhielten eine Monotherapie, Colistin wurde überwiegend (82 %) als Kombinationstherapie verabreicht. Die 28-Tage-Gesamtmortalität lag bei 57 % ohne Unterschiede zwischen den Gruppen (Cefiderocol 55 % vs. Colistin 58 %, *p* = 0,70). In der multivariablen Analyse war der SOFA-Score der unabhängige Risikofaktor für die Sterblichkeit (HR 1,24; 95 %-KI 1,15–1,38; *p* < 0,001; [17]). Cefiderocol war mit einem nichtsignifikant niedrigeren Mortalitätsrisiko verbunden (HR 0,64; 95 %-KCI 0,38–1,08; *p* = 0,10; [17]).

## Fazit für die Praxis

Die Studienergebnisse sowie die Ergebnisse aus dem weltweiten Compassionate-Use-Programm und dem klinischen Alltag weisen darauf hin, dass Cefiderocol eine vielversprechende Behandlungsoption für schwere Infektionen durch multiresistente, insbesondere carbapenemresistente gramnegative Bakterien (Enterobacterales, *Pseudomonas aeruginosa* und *Acinetobacter baumannii*) ist, wenn nur begrenzte Behandlungsoptionen zur Verfügung stehen [5, 26]. Das In-vitro-Wirkspektrum von Cefiderocol umfasst alle wichtigen aeroben gramnegativen Erreger einschließlich der WHO(„World Health Organisation“)-Problemkeime. Vorteile sind auch die gute Verträglichkeit und das geringe Interaktionspotenzial. Der Einsatz sollte in erster Linie als gezielte Therapie erfolgen**.**
